# Supplementary material for: Factors regarding the dog owner’s household situation, antisocial behaviours, animal views and animal treatment in a population of dogs confiscated after biting humans and/ or other animals
Source: PLoS One. 2023 Mar 22;18(3):e0282574. doi: 10.1371/journal.pone.0282574 (PMC10032511; doi:10.1371/journal.pone.0282574)
Supplement: S1 File — (DOCX) [file pone.0282574.s001.docx]

**Supporting Information**

**S1 Appendix – Pairwise comparisons on dog owners’ control situation with regard to the dog, at biting incidents leading to confiscation of 374 dogs**

**S2 Appendix – Severeness of biting incidents according to Ian Dunbar for human/ animal-directed biting incidents caused by 374 confiscated dogs**

**S3 Appendix – The distribution of multifactorial situations of dog ownership factors applying to 374 cases of confiscated dogs**

**S1 Appendix – Pairwise comparisons on dog owners’ control situation with regard to the dog, at biting incidents leading to confiscation of 374 dogs**

Counts for dog owners’ control situation with regard to the dog, at biting incidents leading to confiscation of 374 dogs, for a first time frame (2008-2010), a second time frame (2020-mid-May 2022) and overall for both time frames. We present Chi-square adjusted residuals between brackets and significant deviations from expected values (i.e. ≥│2│, in bold).

| **Control issue** | **2008-2010** | **2020-2022** | **Both time frames** | **χ2, df, P-value** |
| --- | --- | --- | --- | --- |
| Roaming dog | 105 **(-2.4)** | 166 **(2.4)** | 271 | χ2=5.7, df=1, P=0.02 |
| Pulled loose | 11 (-1.5) | 25 (1.5) | 36 | χ2=2.3, df=1, P=0.13 |
| On leash | 34 (**3.4**) | 19 (**-3.4**) | 53 | χ2=11.8, df=1, P=0.001 |
| No information available | 9 (1.7) | 5 (-1.7) | 14 | χ2=2.8, df=1, P=0.09 |
| *Total* | 159 | 215 | 374 |  |

**S2 Appendix – Severeness of biting incidents according to Ian Dunbar for human/ animal-directed biting incidents caused by 374 confiscated dogs**

The severeness of dog bites, indicated based on Ian Dunbar’s biting scale did not differ significantly between a first time frame (2008-2010), a second time frame (2020-mid-May 2022; χ2=1.3, P=0.51, df=2 for human-directed bites and χ2=0.6, P=0.74, df=2 for animal-directed bites).

|  | **Human-directed** | |  | | **Dog-directed** | |  | |
| --- | --- | --- | --- | --- | --- | --- | --- | --- |
|  | 2008-2010 | 2020-2022 | | Both time frames | 2008-2010 | 2020-2022 | | Both time frames |
| **Not mentioned/ applicable** | 57 | 93 | | 150 | 83 | 100 | | 183 |
| **Biting scale 1,2,3**  **(up to four teeth punctures, none deeper than half the length of the dog’s teeth)** | 32 | 35 | | 67 | 6 | 14 | | 20 |
| **Biting scale 3 or 4, 4, 3 or 4 or 5**  **(at least one puncture deeper than half the length of the dog’s teeth, but not multiple bites)** | 35 | 39 | | 74 | 10 | 11 | | 21 |
| **Biting scale 4 or 5, 4 or 5 or 6, 5, 6**  **(multiple bites with at least one puncture deeper than half the length of the dog’s teeth and more severe, including death)** | 35 | 48 | | 83 | 60 | 90 | | 150 |

**S3 Appendix – The distribution of multifactorial situations of dog ownership factors applying to 374 cases of confiscated dogs**

We present the multifactorial situations distribution (median, range: 3, 0-16) for the counts (percentages) on dog ownership factors applying to 374 cases of dogs confiscated after a biting incident, for a first time frame (2008-2010), a second time frame (2020-mid-May 2022) and overall for both time frames.

| **Number of factors reported on for the dog's situation** | **2008-2010** | **2020-2022** | **Both time frames** |
| --- | --- | --- | --- |
|  | **N (% of column total)** | **N (% of column total)** | **N (% of column total)** |
| None | 19 (12%) | 13 (6%) | 32 (9%) |
| One | 37 (23%) | 31 (14%) | 68 (18%) |
| Two | 30 (19%) | 37 (17%) | 67 (18%) |
| Three | 26 (16%) | 22 (10%) | 48 (13%) |
| Four | 22 (14%) | 31 (14%) | 53 (14%) |
| Five | 7 (4%) | 31 (14%) | 38 (10%) |
| Six | 9 (6%) | 12 (6%) | 21 (6%) |
| Seven | 4 (3%) | 12 (6%) | 16 (4%) |
| Eight | 3 (2%) | 5 (2%) | 8 (2%) |
| Nine | 1 (1%) | 1 (1%) | 10 (3%) |
| Ten | 0 (0%) | 0 (0%) | 1 (0%) |
| Eleven | 0 (0%) | 0 (0%) | 3 (1%) |
| Twelve | 0 (0%) | 0 (0%) | 1 (0%) |
| Thirteen | 1 (1%) | 1 (1%) | 2 (1%) |
| Fourteen | 0 (0%) | 4 (2%) | 4 (1%) |
| Fifteen | 0 (0%) | 0 (0%) | 0 (0%) |
| Sixteen | 0 (0%) | 2 (1%) | 2 (1%) |
| Total | 159 | 215 | 374 |
